# 14-3-3ζ Mediates Tau Aggregation in Human Neuroblastoma M17 Cells

**DOI:** 10.1371/journal.pone.0160635

**Published:** 2016-08-22

**Authors:** Tong Li, Hemant K. Paudel

**Affiliations:** 1 The Bloomfield Center for Research in Aging, Lady Davis Institute for Medical Research, Jewish General Hospital, Montreal, Canada; 2 The Department of Neurology and Neurosurgery, McGill University, Montreal, Canada; USF Health Morsani College of Medicine, UNITED STATES

## Abstract

Microtubule-associated protein tau is the major component of paired helical filaments (PHFs) associated with the neuropathology of Alzheimer’s disease (AD). Tau in the normal brain binds and stabilizes microtubules. Tau isolated from PHFs is hyperphosphorylated, which prevents it from binding to microtubules. Tau phosphorylation has been suggested to be involved in the development of NFT pathology in the AD brain. Recently, we showed that 14-3-3ζ is bound to tau in the PHFs and when incubated *in vitro* with 14-3-3ζ, tau formed amorphous aggregates, single-stranded straight filaments, double stranded ribbon-like filaments and PHF-like filaments that displayed close resemblance with corresponding ultrastructures of AD brain. Surprisingly however, phosphorylated and non-phosphorylated tau aggregated in a similar manner, indicating that tau phosphorylation does not affect *in vitro* tau aggregation (Qureshi *et al* (2013) Biochemistry 52, 6445–6455). In this study, we have examined the role of tau phosphorylation in tau aggregation in cellular level. We have found that in human M17 neuroblastoma cells, tau phosphorylation by GSK3β or PKA does not cause tau aggregation, but promotes 14-3-3ζ-induced tau aggregation by destabilizing microtubules. Microtubule disrupting drugs also promoted 14-3-3ζ-induced tau aggregation without changing tau phosphorylation in M17 cell. *In vitro*, when incubated with 14-3-3ζ and microtubules, nonphosphorylated tau bound to microtubules and did not aggregate. Phosphorylated tau on the other hand did not bind to microtubules and aggregated. Our data indicate that microtubule-bound tau is resistant to 14-3-3ζ-induced tau aggregation and suggest that tau phosphorylation promotes tau aggregation in the brain by detaching tau from microtubules and thus making it accessible to 14-3-3ζ.

## Introduction

Neurofibrillary tangles (NFTs) are one of the characteristic pathogenic lesions found in the brains of patients suffering from Alzheimer’s disease (AD) [1, 2]. NFTs are composed mainly of paired helical filaments (PHFs), with tau protein being the major structural component of PHFs. In the normal brain, tau provides structural stability to neurons by stabilizing the microtubule cytoskeleton. Tau isolated from PHFs is hyperphosphorylated and does not bind to microtubules [3]. In AD brain, tau hyperphosphorylation is thought to precede and promote tau aggregation [3]. It has been suggested that tau hyperphosphorylation destabilizes microtubules and causes neurodegeneration and is a critical event in the development of NFT pathology [4, 5]. However, *in vitro*, tau phosphorylation alone does not cause tau aggregation. Instead, nonphosphorylated tau aggregates when incubated with a number of acidic molecules (see below).

Tau is a soluble molecule but aggregates when incubated with a number of small polyanions such as heparin, polyglutamate, RNA, DNA, and fatty acids *in vitro* [6–10]. All of these molecules bind to the microtubule-binding region of tau and compete with microtubules for tau binding [8, 11]. Moreover, the PHF core contains the microtubule-binding repeats of tau [12]. *In vitro*, tau fragments derived from microtubule-binding repeats are significantly more prone to aggregation than the full-length tau [8]. These observations suggest that the microtubule-binding region of tau is involved in tau aggregation and microtubule bound tau may be resistant to aggregation.

14-3-3 proteins regulate a wide variety of physiological processes such as cell division, cell differentiation, apoptosis, and signal transduction. There are seven 14-3-3 isoforms ß,γ,ξ,τ,η,σ and ζ (α and ∂ are phosphorylated forms of ß and ζ) [13]. Among these isoforms, 14-3-3ζ was found to be associated with amyloid plaques [14] and PHFs [15] and upregulated in AD brain [16, 17]. *In vitro*, 14-3-3ζ binds to tau and promotes tau phosphorylation and aggregation [18–21]. Recently, we showed that 14-3-3ζ is bound to tau within the PHFs of AD brain [15]. *In vitro* incubation of tau with 14-3-3ζ resulted in the formation of PHF-like filaments. Our results and data from previous studies suggest that 14-3-3ζ causes tau aggregation during the development of NFT pathology. 14-3-3ζ-induced tau aggregation is being used as a model to study mechanism of tau aggregation to PHFs [15, 18, 20–23]. Surprisingly, we found that *in vitro* both phosphorylated and nonphosphorylated tau aggregated in the presence of 14-3-3ζ in a similar manner and that tau phosphorylation did not affect tau aggregation [15]. Since, tau in PHFs is always hyperphosphorylated and tau hyperphosphorylation is thought to promote tau aggregation during PHF formation, [2, 3, 5], these observations have raised a question regarding the role of tau phosphorylation in 14-3-3ζ-induced tau aggregation in AD brain.

To answer the above question, we have examined tau aggregation in human BE(2)-M17 neuroblastoma cells. These cells have been used extensively as *in vitro* model for studies on neuronal development, neurological diseases and mechanism of action and are relative easy to handle and amenable to gene transfection [24, 25]. More importantly, they express tau and 14-3-3ζ and hence provide a good cell model to study tau function and aggregation in intact cells. Herein we report that tau forms amorphous aggregates when co-expressed with 14-3-3ζ in these cells. Interestingly, and in contrast with our *in vitro* data, tau phosphorylation by GSK3β or PKA promoted 14-3-3ζ-induced tau aggregation. By using *in vitro* microtubule sedimentation assay, we demonstrate that microtubule-bound tau is resistant to 14-3-3ζ-induced aggregation and that tau phosphorylation promotes its aggregation by inhibiting tau from binding to microtubules, thus making it accessible to 14-3-3ζ. Our data provides a novel mechanism for tau aggregation in the AD brain.

## Materials and Methods

### cDNA cloning, Cell culture, Transfection and Drug treatment

Flag-tau, Myc-14-3-3ζ and HA-GSK3β cDNA clones used are described previously [26]. pcDNA3 plasmid expressing Myc-PKA was a gift from Dr. Dong Han of McGill University. Human M17 neuroblastoma cells were cultured and transfected using Lipofectamine 2000 (Invitrogen Burlington, ON, Canada) [27]. Solutions of colchicine, and nocodazole (all from Sigma-Aldrich, Oakville, ON, Canada) were freshly prepared and diluted in culture medium. Cells transfected with indicated genes for 48 hr were treated with each drug for 2 hr. The final concentration of colchicine and nocodazole were 0.05 and 0.2 μg/ml, respectively.

### Proteins and antibodies

Tau was purified from bacterial extract expressing the longest isoform of human tau [28]. GST-14-3-3ζ and GST were purified from bacterial extract using glutathione sepharose affinity chromatography [29]. Purified GST-14-3-3ζ was treated with precision protease (Sigma-Aldrich, Oakville, ON, Canada) to separate GST and 14-3-3ζ. The treated sample was chromatographed through a glutathione sepharose column [29]. Monoclonal anti-HA, anti-Myc and anti-Flag, as well as monoclonal and polyclonal anti-14-3-3ζ and anti-tau antibodies have been described previously [26]. Monoclonal antibodies against Ac-Tub and Tyr-Tub were obtained from Sigma-Aldrich (Oakville, ON, Canada). Tau phosphorylation-specific antibodies PHF1 and pS214 were also described previously [30]. The concentration of tau protein was determined by a spectrophotometer as described previously by using E_280_ value of 2.8 for 1% protein [31]. The concentration of phosphorylated tau was determined by the BioRad protein assay (BioRad Canada, Mississauga, ON) using tau as the standard. Concentrations of 14-3-3ζ and all other proteins were determined by the BioRad protein assay using BSA as the standard.

### Preparation of phosphorylated tau

Tau was phosphorylated to 7.9 mol of phosphate /mol of tau via incubation with fresh rat brain extract in a mixture containing 1 mg/ml of tau, 25 mM HEPES (pH 7.2), 0.1 mM EDTA, 0.1 mM DTT, 10 mM NaF, 50 mM β-glycerol phosphate, 0.5 mM [γ^32^p]ATP, 10 mM MgCl_2_, 0.1 mM CaCl_2_, phosphatase and protease inhibitor cocktail (Roche Canada, Toronto, ON), and carryover amounts of brain extract and was purified through a Sephadex G25 column as described previously [15].

### Immunocytochemistry

Immunocytochemistry was performed as described previously [30]. Briefly, cells grown on coverslips to ~80% confluency and fixed with 4% paraformaldehyde were incubated with 0.05% thioflavin S (Sigma-Aldrich, Oakville, ON, Canada) for 8 min and washed with 80% ethanol three times for 5 min each. Washed cells were permeabilized via incubation with 0.1% triton X-100 and then incubated with anti-Myc (Myc-14-3-3ζ) or anti-Flag (Flag-tau) anti-body for 12 hr at 4°C. Incubated cells were washed and then developed with Cy3-conjugated second antibody and visualized under the fluorescent microscope. To develop S1 Fig, Alex Fluor 488 and Cy3-conjugated second antibodies were used.

### Quantitative centrifugation assay of tau aggregation

Tau aggregation was monitored and quantified by a method previously described [32]. Cells were lysed in lysis buffer (50 mM Tris-HCl (pH 7.4), 150 mM NaCl, 100 mM β-glycerophosphate, 10 mM EDTA, 10 mM EGTA, 10 mM NaF, 10 mM MgCl_2_ and 0.2% Nonidet P-40) containing a protease and phosphatase inhibitor cocktail. Each lysate (100 μl) was centrifuged at 100,000 x g for 1 h at 4°C. The supernatant and the pellet were separated. The pellet was washed with lysis buffer and dissolved in 100 μl of SDS/PAGE sample buffer. An aliquot (25 μl each) of the supernatant and the pellet were subjected to Western blot analysis. Based on band intensities, the relative amount of each protein in the pellet and the supernatant was calculated. The relative amount of protein in the pellet was regarded as the aggregated protein and is expressed as % of the total (amount in the pellet + that in the supernatant).

### Western blotting

Proteins were separated on SDS-PAGE and then Western blotted. Immunoreactivity was detected by using enhanced chemiluminescence reagent followed by exposure to X-ray film. Films were scanned densitometrically and intensities of bands were quantified using Image J software (NIH).

### Immunoelectron microscopy

Immunoelectron microscopy (Immuno EM) was performed as described previously [15]. Each sample was adsorbed on a 300 mesh carbon-coated copper grid. After 5 washes, each grid was blocked with 2% BSA followed by exposure to anti-Tau-5 (total tau) or PHF-1 (phosphorylated tau) monoclonal antibody. After 2 h of exposure followed by 5 washes, each grid was incubated with anti-14-3-3ζ polyclonal antibody for 2 h. Grids were washed and sequentially labeled with anti-rabbit followed by anti-mouse second antibodies conjugated to 10 and 18 nm colloidal gold, respectively. After washing, samples were negatively stained with urinyl acetate and viewed under the EM.

### Microtubule sedimentation assay

Microtubule sedimentation assay was performed as described previously [33] with some modifications. Assembly-competent tubulin was purified from porcine brain [34]. To perform the sedimentation assay, stock solution of purified tubulin was thawed in ice. To a vial containing tau in PIPES buffer (0.1M Pipes, (pH 6.6), 1 mM EGTA, 1 mM MgSO_4_ and 1 mM β-mercapotethanol) supplemented with protease inhibitor cocktail, an aliquot of GTP, and taxol were added and the vial was placed in a water bath maintained at 37°C. Tubulin was added to the vial and after 30 min of incubation, 14-3-3ζ or GST was added to the mixture and the incubation was continued at a lower temperature of 25°C. The final volume of the mixture was 100 μl and the concentrations of various components of the mixture were 100 mM Pipes, 1 mM GTP, 1 mM MgSO_4_, 1 mM β-mercaptoethanol, 10 μM taxol, 3 mg/ml of tubulin, 0.5 mg/ml of tau, and 0.5 mg/ml of 14-3-3ζ or 0.5 mg/ml of GST. After 48 h, the samples were centrifuged at 30,000 x g for 30 min. The supernatant (S1) and the pellet (P1) were separated and the pellet was dispersed in 100 μl of cold microtubule disassembly buffer (PIPES buffer containing 3 mM CaCl_2_). Dispersed pellet was incubated in ice for 30 min to allow microtubules to depolymerize and then centrifuged at 100,000 x g for 30 min at 4°C. The supernatant (S2) and the pellet (P2) were separated. Pellet P2 was dispersed in 25 μl Pipes buffer and 5 μl was analyzed by EM. Equal volumes of supernatant and pellet were analyzed by SDS-PAGE.

### Density Gradient Ultracentifugation

Stock OptiPrep medium (60%) (Sigma-Aldrich) was diluted to 50, 40, 30, 20, or 10% using distilled H_2_O and 900 μl of each diluted medium was layered gently into a 5 ml ultracentrifuge tube in a descending concentration from 50 to 10%. Cell lysate (500 μl each) was layered onto the top of the gradient and the tubes were centrifuged at 145,000 x g for 12 h in a SW 55 Ti rotor at 4°C. After centrifugation, fractions (500 μl each) were withdrawn from the top and 25 μl of each fraction was analyzed by Western blotting.

### Immunoprecipitation

Immunoprecipitation was carried out as described previously [29]. Briefly, 200 μl of fraction from density gradient centrifugation was pre-cleared with protein G agarose beads and then mixed with 10 μl of either anti-tau, anti-14-3-3ζ antibody or IgG and then incubated at 4°C over night. Antibodies were captured by adding 30 μl of protein G agarose beads. Beads were washed and then analyzed by Western blotting.

### Statistics

The data was analyzed by one-way or two-way ANOVA followed by Bonferroni’s *post hoc* test for multigroup and the student’s t-test for two group comparisons and is expressed as the mean ± SEM with *p*<0.05 considered significant.

## Results

### 14-3-3ζ promotes tau aggregation in human M17 neuroblastoma *cells*

**Fig 1:**
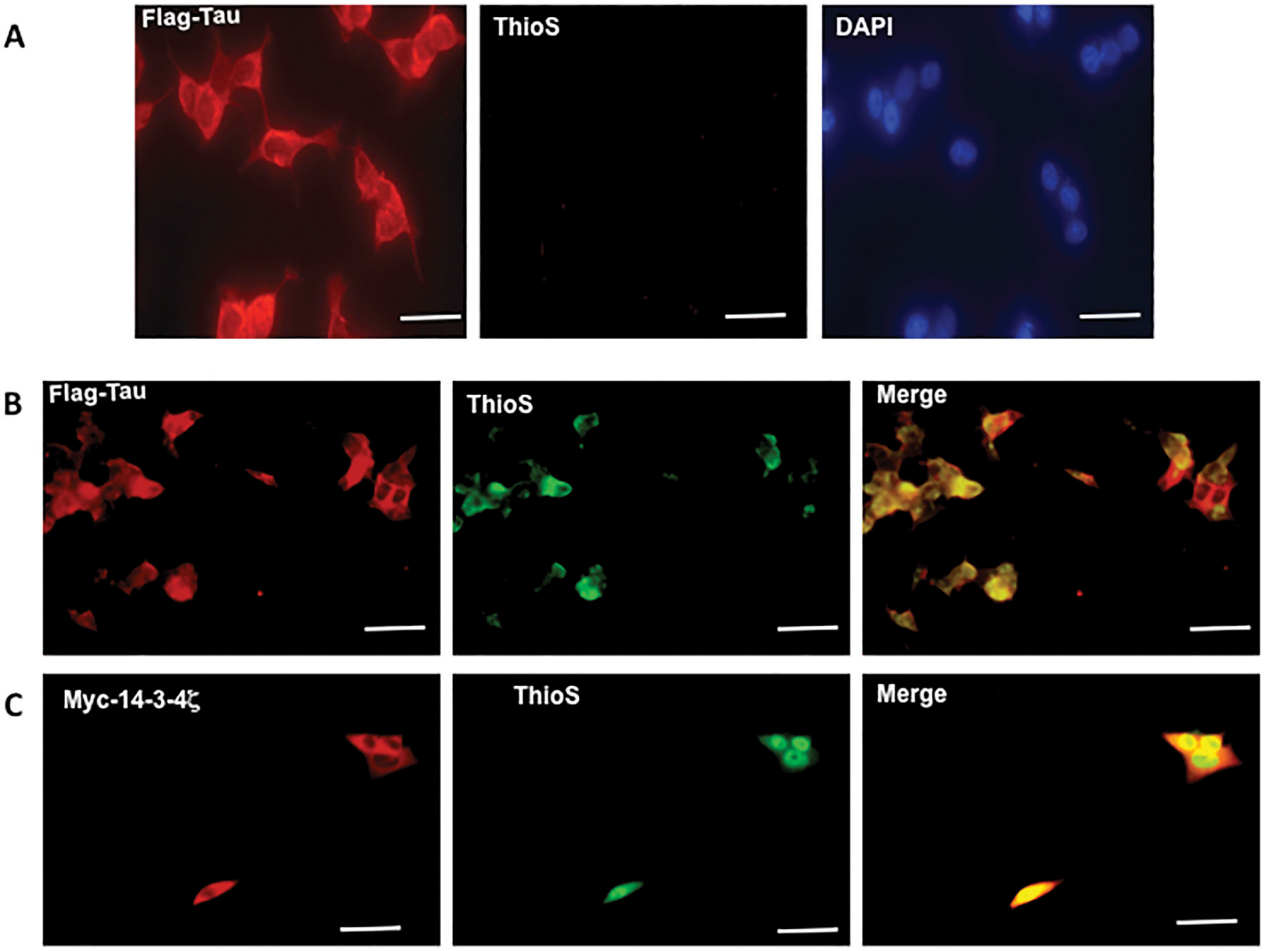
Co-expression of 14-3-3ζ and tau in human neuroblastoma cells causes formation of cytoplasmic thioflavin S positive inclusions. Cells transfected with either Flag-tau or Flag-tau and Myc-14-3-3ζ were fixed and then stained with thioflavin S (green) followed by either anti-Flag for tau (red) or anti-Myc for 14-3-3ζ (red). *A*, cells transfected with tau alone stained for tau (red), Thioflavin S (green) and DAPI (nucleus). *B* and *C*, cells co-transfected with Flag-tau and Myc-14-3-3ζ. Scale bars 40 μM (*A*); 100 μM (*B* and *C*).

When, human M17 neuroblastoma cells co-transfected with the longest isoform of human tau and human 14-3-3ζ were examined under the fluorescent microscope, tau was cytoplasmic but 14-3-3ζ was localized in both cytoplasm and nucleus. Colocalization of both protein was observed in the cytoplasm (S1 Fig, yellow). In a previous study, we showed that 14-3-3ζ and tau co-imunoprecipitate from lysates of HEK-293 cells transfected with 14-3-3ζ and tau [26]. Thus, as seen in HEK-293 cells [26], 14-3-3ζ and tau associate when co-expressed in M17 cells. To evaluate the effect of 14-3-3ζ on tau aggregation, we stained transfected cells with thioflavin S, which stains aggregated proteins. In cells expressing tau alone, the thioflavin S staining was below the detection limit (Fig 1*A*). Cells expressing tau and 14-3-3ζ, on the other hand, displayed intense cytoplasmic thioflavin staining (Fig 1*B*, green) that co-localized with tau (Fig 1*B*, yellow) and surrounded by 14-3-3ζ (Fig 1*C*, yellow).

**Fig 2:**
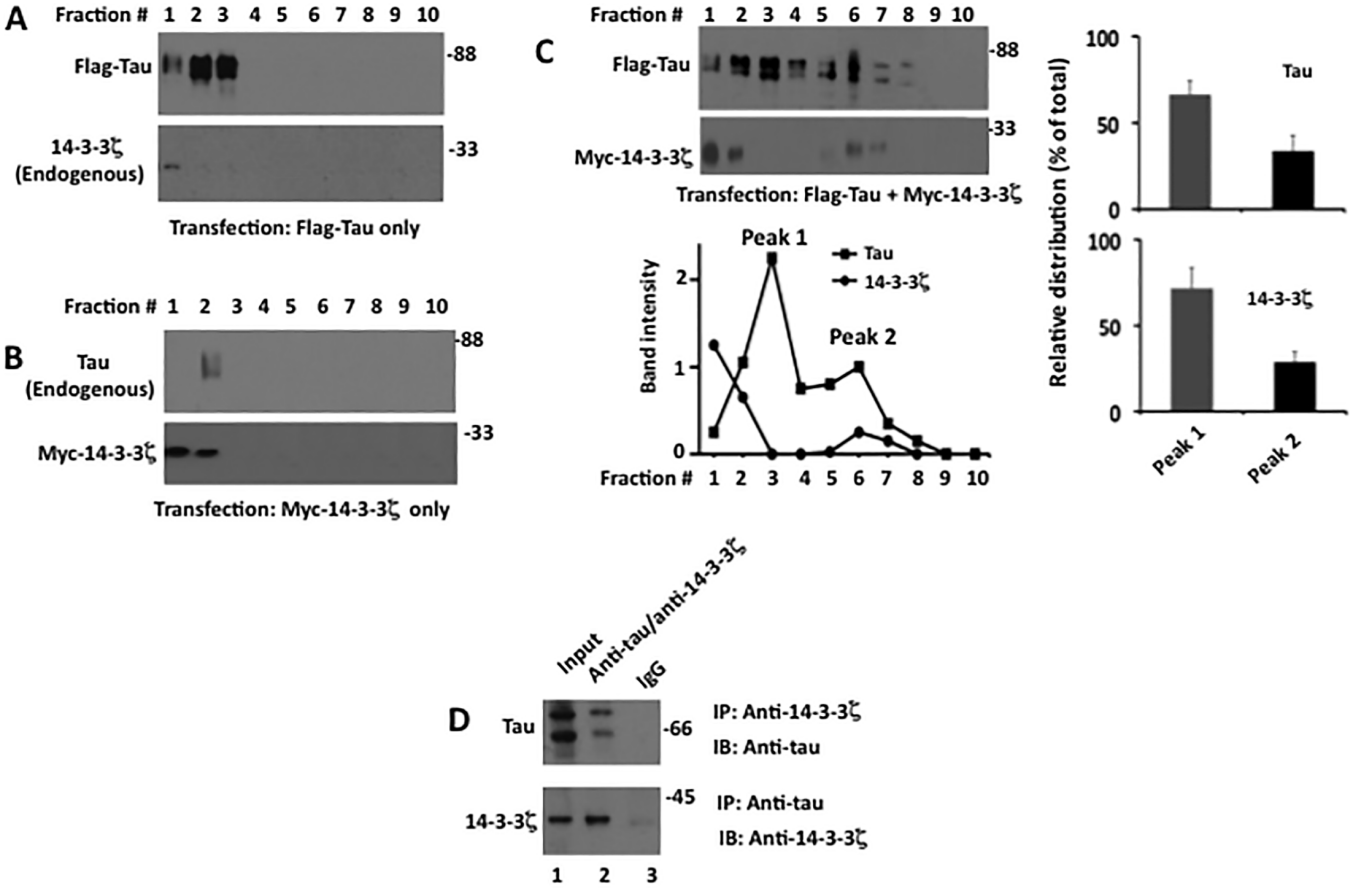
Tau and 14-3-3ζ forms a high molecular weight complex when co-expressed in M17 human neuroblastoma cells. Cells transfected with either Flag-tau or Myc-14-3-3ζ or co-transfected with Flag-tau and Myc-14-3-3ζ were subjected to OmniPrep density gradient centrifugation. Fractions were collected and analyzed by Western blotting and immunoprecipitation. *A*, Western blot of fractions corresponding to cells transfected with Flag-tau alone. *B*, Western blot of fractions corresponding to cells transfected with Myc-14-3-3ζ alone. *C*, Western blot of samples corresponding to cells co-transfected with Flag-tau and Myc-14-3-3ζ. Based on blot band intensities, the relative amount of Peak 1 (sum of fractions 1–4) and peak 2 (sum of fractions 5–8) was calculated and is expressed as the % of total (sum of fractions 1–8). *D*, Immunoprecipitation. Immunoprecipitation was carried out using fraction # 6 from panel *C*.

In the previous study, we showed that when subjected to sucrose density gradient centrifugation, monomeric soluble tau stays in the upper factions containing lower concentration of sucrose. Aggregated tau, on the other hand, enters the gradient and is recovered in the fraction containing higher sucrose concentration [15]. We further examined Flag-tau and Myc-14-3-3ζ transfected cells first by Western blotting for proteins expression (S2 Fig) and then OptiPrep density gradient ultracentrifugation for protein aggregation (Fig 2). Tau from cells transfected with Flag-tau alone was present in top fractions 1–3 corresponding to low molecular weight species (Fig 2*A*). Likewise, 14-3-3ζ from cells transfected with Myc-14-3-3ζ was recovered from the top of the gradient corresponding to low molecular weight species (Fig 2*B*). Endogenous 14-3-3ζ from cells transfected with Flag-tau or endogenous tau from cells transfected with Myc-14-3-3ζ were also recovered from the top fractions (Fig 2*A* and 2*B*). This data showed that tau and 14-3-3ζ exist as low molecular weight species in cells transfected only with either tau or 14-3-3ζ.

When cells co-transfected with Flag-tau and Myc-14-3-3ζ were analyzed, tau was detected in fractions 1 through fraction 8 forming two peaks (Fig 2*C*). Peak 1 was present within fractions 1–4 and contained 66.3% of total tau that was loaded onto the gradient (Fig 2*C*, right panel). Elution profile of tau in peak 1 was similar to that of the low molecular weight tau of cells transfected with Flag-tau alone. The elution profile of tau in peak 2 on the other hand was of a high molecular weight species and this peak contained 33.7% of total tau that was loaded onto the gradient. When 14-3-3ζ was analyzed, most of it was recovered in top fractions as low molecular weight species (Fig 2*C*). However, 28.6% of total 14-3-3ζ loaded onto the gradient co-eluted with tau in peak 2 (Fig 2*C*). This data indicated that both tau and 14-3-3ζ have formed high molecular weight species with similar weight in cells co-transfected with tau and 14-3-3ζ.

To determine if tau and 14-3-3ζ in peak 2 fractions represent components of the same or different high molecular weight species, we performed a co-immunoprecipitation experiment using fraction #6 from peak 2 (Fig 2*D*). Tau was pulled down by anti-14-3-3ζ antibody as was 14-3-3ζ with tau. Based on this result, we concluded that tau and 14-3-3ζ form a high molecular weight species in cells that are co-transfected. This, in turn, suggested that tau aggregates when co-transfected with 14-3-3ζ in these cells.

**Fig 3:**
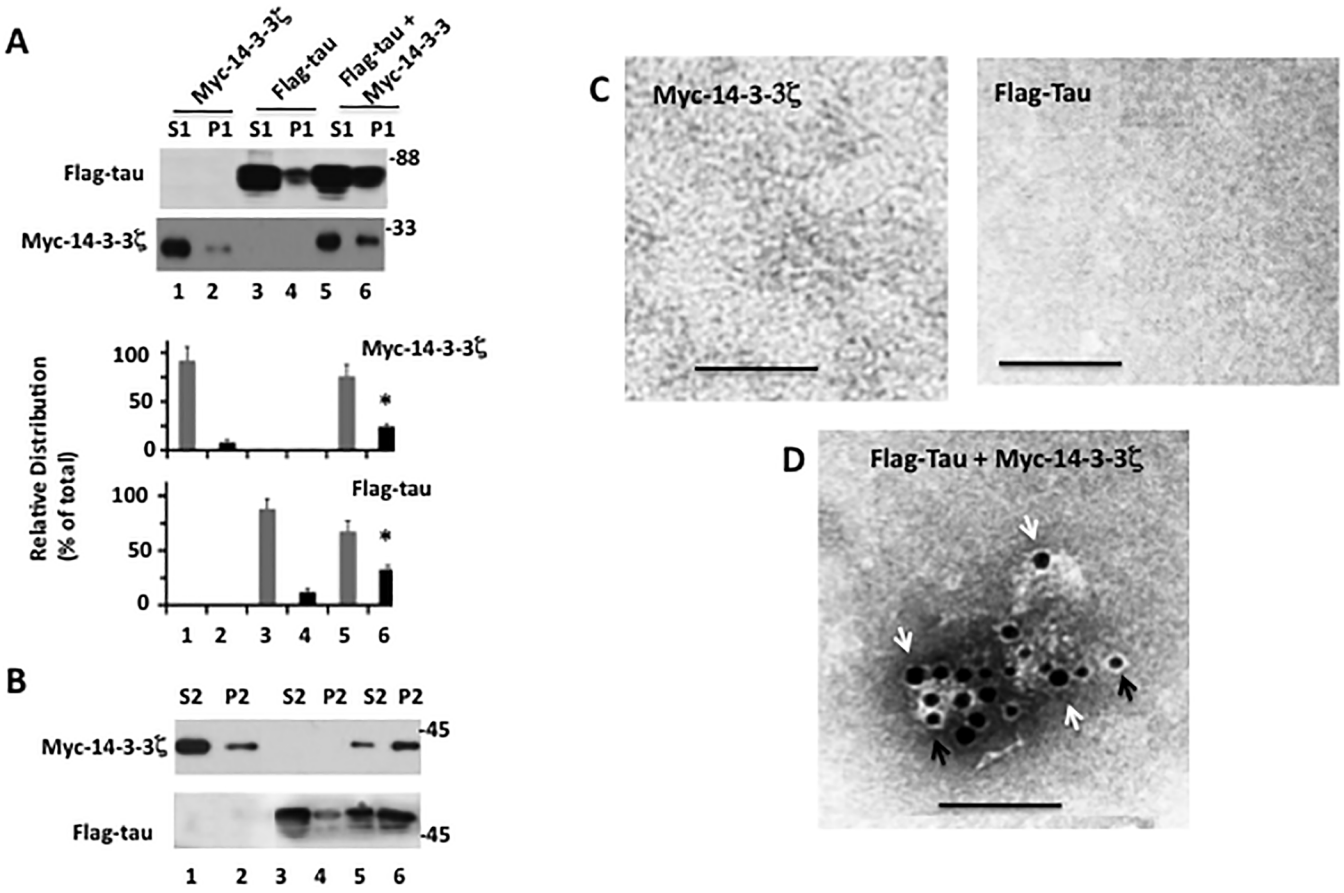
14-3-3ζ promotes tau aggregation in M17 human neuroblastoma cells. M17 human neuroblastoma cells transfected with Myc-14-3-3ζ alone, Flag-tau alone or co-transfected with Flag-tau and Myc-14-3-3ζ (S2 Fig) were subjected to quantitative centrifugation assay for tau aggregation followed by Immuno EM. *A*, Quantitative centrifugation assay. Each cell lysate was centrifuged and separated into soluble supernatant fraction S1 and the insoluble fraction P1. Each P1 fraction was re-suspended in buffer and analyzed by Western blot analysis. Based on the blot band intensity, the relative distribution of each protein in each fraction was calculated. The relative distribution is the amount of a protein in the indicated fraction normalized against the total of that protein (sum of S1 and P1). The values with standard error are the average of three experiments. *P< 0.05 with respect to pellet of cells expressing 14-3-3ζ only or Flag-tau only. *B*, Western blot. Each P1 pellet from panel *A* was resuspended in buffer, incubated with constant shaking and re-centrifuged. Resulting soluble S2 and insoluble P2 fraction were analyzed by Western blot analysis. In both *A* and *B* panels, lanes 1 and 2 correspond to cells transfected with Myc-14-3-3ζ alone whereas lanes 3 and 4 correspond to cells transfected with Flag-tau alone. Likewise, lanes 5 and 6 correspond to cells co-transfected with Flag-tau and Myc-14-3-3ζ. *C* and *D*, Immuno EM. Each P2 pellet from panel *B* was analyzed by Immuno EM. Black arrow indicates 10 nm gold particle attached to anti-tau antibody. White arrow indicates 18 nm gold particle attached to anti-14-3-3ζ antibody. Scale bar 100 nm.

To substantiate the above data, we analyzed cells co-transfected with 14-3-3ζ and tau (S2 Fig) for tau aggregation using centrifugation assay (Fig 3*A*). In cells expressing 14-3-3ζ alone, ~8% of the total 14-3-3ζ was present in the pellet (P1) containing insoluble materials (lane 2). Likewise, in cells expressing tau alone, ~12% of total tau was recovered in P1 (lane 4). In the P1 of cells co-expressing tau and 14-3-3ζ on the other hand, the amount of 14-3-3ζ and tau was 2.3 and 2.7-fold more than that of the P1 of cells expressing 14-3-3ζ alone or tau alone, respectively (lane 6). To evaluate if Myc-14-3-3ζ and tau in these pellets were aggregated or in soluble form trapped during centrifugation, each pellet was dispersed in the buffer, incubated with constant shaking for 1 hr at 37°C, centrifuged, and analyzed by Western blotting. Most of 14-3-3ζ and tau in respective control cells expressing 14-3-3ζ alone or tau alone became soluble and were recovered in the supernatant (Fig 3*B*, lanes 1 and 3). In contrast, relatively large amount of 14-3-3ζ and tau in the sample from the cells expressing tau and 14-3-3ζ remained insoluble and were present in the pellet (P2) (Fig 3*C*, lane 6). When viewed under EM, the P2 pellet of the cells expressing either 14-3-3ζ alone or tau alone did not show any ultrastructure (Fig 3*C*). The P2 pellet of cells co-expressing tau and 14-3-3ζ on the other hand, displayed large amorphous aggregates that were decorated with both anti-tau (black arrow) and anti-14-3-3ζ (white arrow) gold particles (Fig 3*E*). Based on this data, we concluded that tau forms amorphous aggregates when co-overexpressed with 14-3-3ζ in human M17 neuroblastoma cells.

### Phosphorylation promotes tau aggregation in human M17 neuroblastoma cells

**Fig 4:**
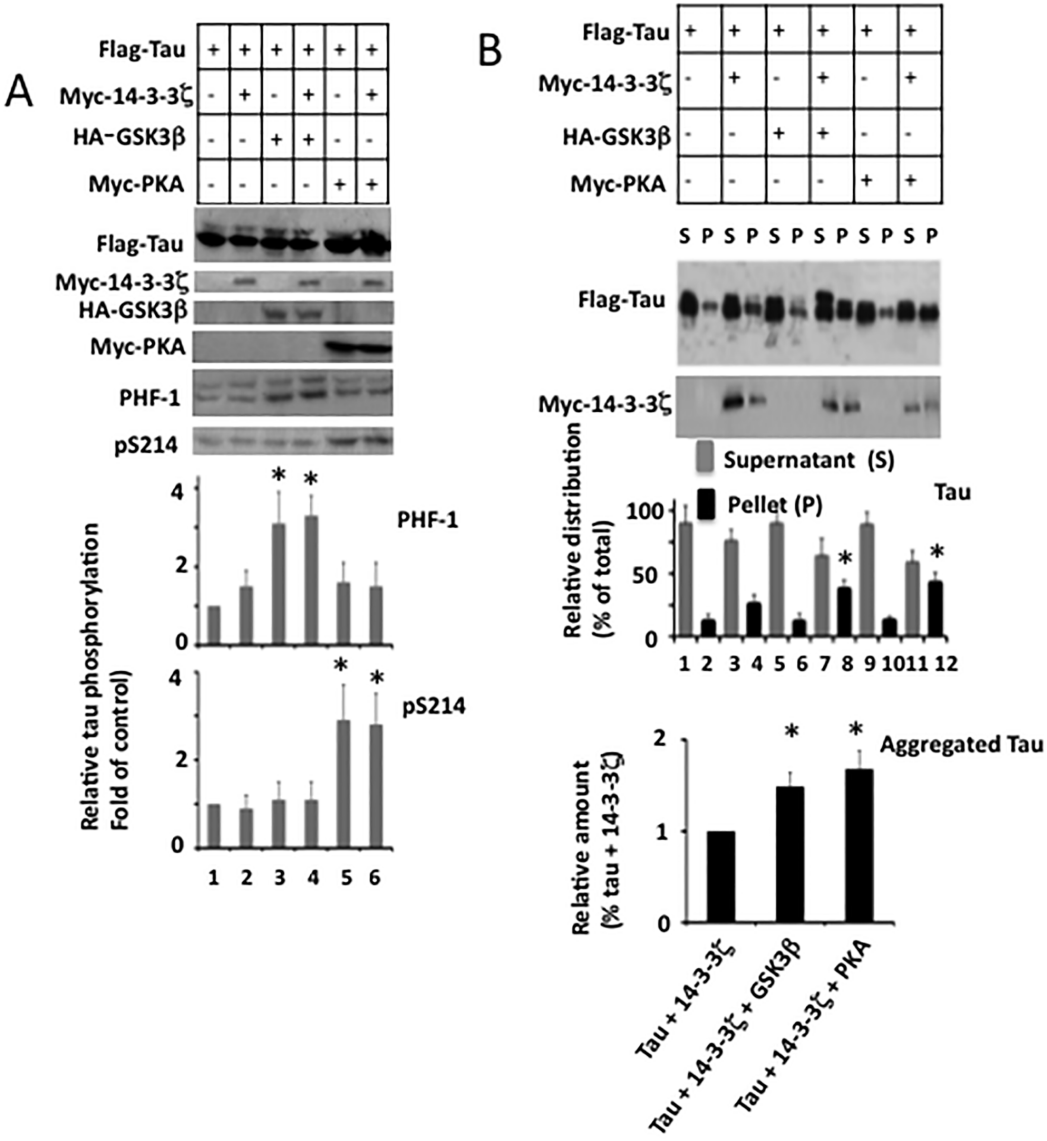
Phosphorylation promotes 14-3-3ζ-induced tau aggregation in M17 human neuroblastoma cells–M17 human neuroblastoma cells co-transfected with Flag-tau, Myc-14-3-3ζ, HA-GSK3β and Myc-PKA in various combinations were analyzed by Western blotting for tau phosphorylation and then by centrifugation assay for tau aggregation. *A*, Western blot analysis. PHF-1 and pS214 blots represent tau phosphorylated at Ser^396/404^ and Ser^214^, respectively. The ser^396/404^ site is phosphorylated by GSK3β whereas the ser^214^ site is phosphorylated by PKA. Based on tau band intensities the relative amount of phosphorylated tau was determined. The relative amount of phosphorylated tau was determined by normalizing the phosphorylated tau band intensity by respective band intensity of total tau. Values with ±SE are the average of three determinations. *P< 0.05 with respect to control cells expressing tau alone. *B*, Centrifugation assay. The centrifugation assay was performed as described in the Materials and Methods. S and P indicate supernatant and pellet, respectively. Based on Flag-tau blot band intensity, the relative distribution of tau and the relative amount of aggregated tau in the indicated fractions were determined as per Fig 2*A*. The relative amount of aggregated tau is expressed as fold of cells expressing tau and 14-3-3ζ. Values in the bar graph are mean ± S.E. and are from three independent experiments. *P < 0.05 with respect to cells expressing tau and 14-3-3ζ.

**Fig 5:**
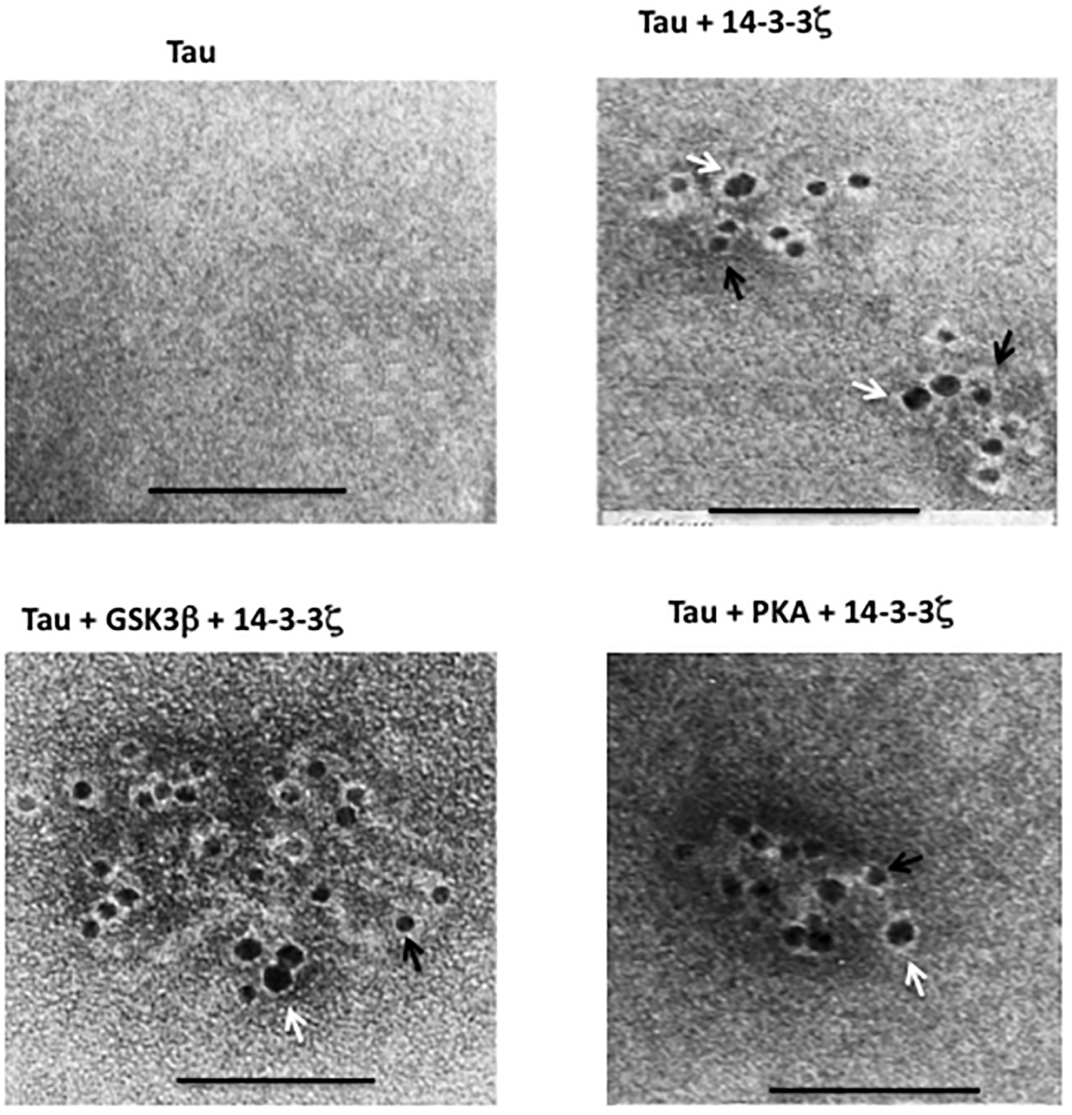
Immuno EM of tau aggregates formed in M17 human neuroblastoma cells expressing Flag-tau, Myc-14-3-3ζ, HA-GSK3β and Myc-PKA in various combination. Pellets obtained by centrifugation assay from Fig 4*B* were analyzed by Immuno-EM as in Fig 3*E*. Scale bar 100 nm.

PHF-tau is phosphorylated at proline-directed and non-proline-directed sites [4, 35]. GSK3β is one of the main proline-directed kinases that phosphorylate tau in the brain [36–38]. Likewise, PKA is one of the major non-proline-directed kinases to phosphorylate tau in the brain [39, 40]. To evaluate the role of phosphorylation in tau aggregation at the cellular level, we co-transfected tau in human M17 neuroblastoma cells with 14-3-3ζ and either GSK3β or PKA. Cell lysates were analyzed for tau phosphorylation using tau phosphorylation-specific antibodies (Fig 4*A*) and tau aggregation by centrifugation assay (Fig 4*B*), which was followed by EM (Fig 5).

In cells transfected with tau and GSK3β, tau phosphorylation at the PHF-1 (Ser^396/404^) site was more than 3-fold higher than basal level observed in cells transfected with tau alone (compare lane 3 or lane 4 with lane 1 in Fig 4*A*). Similarly, in cells transfected with tau and PKA, tau phosphorylation at PKA-specific site Ser^214^ [41] was significantly higher than the basal level (compare lane 5 or lane 6 with lane 1 in Fig 4*A*). Thus, as expected, both GSK3β and PKA phosphorylated tau at their respective sites in M17 cells.

When lysates were analyzed for tau aggregation, the relative amount of aggregated tau in the pellet of sample containing tau and GSK3β or tau and PKA was similar to the basal level that was observed in cells expressing tau alone (compare lane 6 or lane 10 with lane 2 in Fig 4*B*). Thus, tau phosphorylation by either GSK3β or PKA did not promote tau aggregation in M17 cells. However, in cells co-expressing tau and 14-3-3ζ, aggregated tau in the pellet fraction was increased by 2.2-fold when compared to cells expressing tau alone (Fig 4*B*, lane 4). Interestingly, there was a 3.2-fold increase in aggregated tau in cells expressing tau, 14-3-3ζ and GSK3β (lane 8); and 3.7-fold increase in cells expressing tau, 14-3-3ζ and PKA (Fig 4*B*, lane 12). More importantly, compared to cells expressing tau and 14-3-3ζ, cells expressing tau, 14-3-3ζ, and GSK3β contained a 1.5-fold greater amount of aggregated tau (Fig 4*B*, lower panel). Likewise, cells expressing tau, 14-3-3ζ, and PKA contained a 1.7-fold greater amount of aggregated tau than those expressing tau and 14-3-3ζ (Fig 4*B*, lower panel). Under the EM, the pellet of control cells expressing only tau did not contain any aggregated tau (Fig 5), but each pellet of samples expressing tau and 14-3-3ζ; tau, 14-3-3ζ, and GSK3β; and tau, 14-3-3ζ, and PKA displayed anti-tau (black arrow) and anti-14-3-3ζ (white arrow) co-labeled amorphous aggregates (Fig 5). This result demonstrated that tau phosphorylation by GSK3β or PKA enhances 14-3-3ζ -induced tau aggregation in M17 cells.

### Effect of microtubules on tau aggregation

Recently, we showed that tau phosphorylation does not affect 14-3-3ζ-induced tau aggregation *in vitro* [15]. In M17 cells however, tau phosphorylation by GSK3β or PKA promoted 14-3-3ζ-induced tau aggregation (Figs 4 and 5). This data suggested that 14-3-3ζ causes tau aggregation *in vitro* and in M17 cells via different mechanisms. Alternatively, in the previous study, tau was incubated with 14-3-3ζ in the absence of any other proteins [15]. In M17 cells, on the other hand, the tau-14-3-3ζ interaction occurred in the presence of other cellular proteins that bind to tau and 14-3-3ζ. It is possible that any of these proteins may have influenced the interaction of 14-3-3ζ with tau and thus tau aggregation. Tubulin, the major building block of microtubules, is ubiquitously expressed in all cells and is one of the major tau binding proteins in neurons. Moreover, tau binds to and stabilizes microtubules [2], and this binding is negatively affected by tau phosphorylation including at Ser^396^ and Ser^214^ [28, 42, 43]. Therefore, to test the 2^nd^ mechanism, we analyzed microtubules in the lysates of the transfected cells used to generate Fig 4*A*. Acetylated tubulin (Ac-Tub) was used to represent stable microtubules and tyrosinated tubulin (Tyr-Tub) was used to represent unstable microtubules.

**Fig 6:**
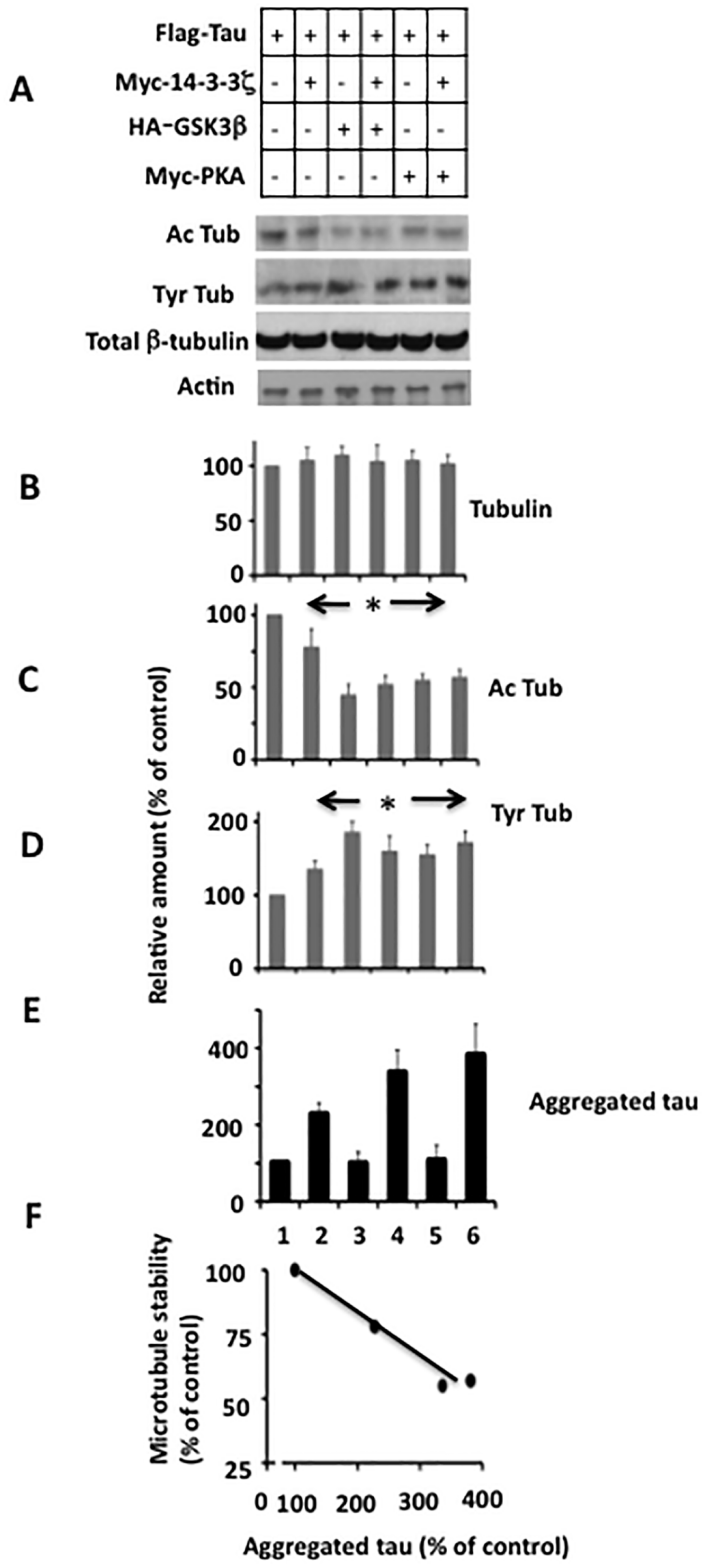
Inverse correlation between microtubule stability and 14-3-3ζ-induced tau aggregation in M17 human neuroblastoma cells. Samples from Fig 4*A* were analyzed by Western blot analysis for microtubules. The relative amount of tubulin was calculated from normalizing the tubulin band of each sample with the corresponding actin band of that sample. Likewise, the relative amounts of Ac-Tub (Ac-tubulin) and Tyr-Tub (Tyr-tubulin) were calculated by normalizing Ac-Tub band or Tyr-Tub band with corresponding β-tubulin band. The relative amount of aggregated tau is from panel *B* of Fig 4. Values in the bar graph are the average ± S.E. of three independent experiments. *p< 0.05 against the cells transfected with Flag-tau alone. *F*, Correlation. Data for microtubule stability and aggregated tau are from panel *C* and *E*, respectively. Plot was generated by using values from lanes 1, 2, 4 and 6 only.

As shown in Fig 6*E*, a significant amount of aggregated tau was observed only in those cells that co-expressed tau and 14-3-3ζ (lanes 2, 4 and 6). In each of these cells, the relative amount of Ac-Tub was lower (Fig 6*C*), while Tyr-Tub level was higher than the basal level (Fig 6*D*). Moreover, in cells expressing tau and 14-3-3ζ, the relative amount of Ac-Tub was 22% lower than the basal level (lane 2). In these cells, the amount of aggregated tau increased by 2.2-fold when compared to the basal level (lane 2). In cells expressing tau, GSK3β, and 14-3-3ζ, microtubule stability decreased by 55% and tau aggregation increased by 3.3-fold (compare lane 1 and lane 4). Likewise, in cells expressing tau, 14-3-3ζ, and PKA, microtubule stability decreased by 43% and tau aggregation was increased by 3.7-fold when compared to the basal levels (compare lane 1 with lane 6). This observation is consistent with 2^nd^ mechanism and suggested an inverse correlation between microtubule stability and 14-3-3ζ-induced tau aggregation in M17 cells (Fig 6*F*).

### Microtubule disruption promotes 14-3-3ζ-induced tau aggregation in human neuroblastoma M17 cells

Tau phosphorylation reduces tau affinity for microtubules and thus causes microtubule instability [4]. Indeed, the relative amount of Ac-Tub was reduced by several folds compared to the basal level in cells that expressed tau and either GSK3β or PKA, in which tau was phosphorylated (Fig 6*B* and 6*C*). As discussed above, tau phosphorylation promotes tau aggregation only in the presence of 14-3-3ζ (Fig 6*E*, lanes 4 and 6). These results suggested that tau phosphorylation might promote 14-3-3ζ-induced tau aggregation indirectly by destabilizing microtubules.

**Fig 7:**
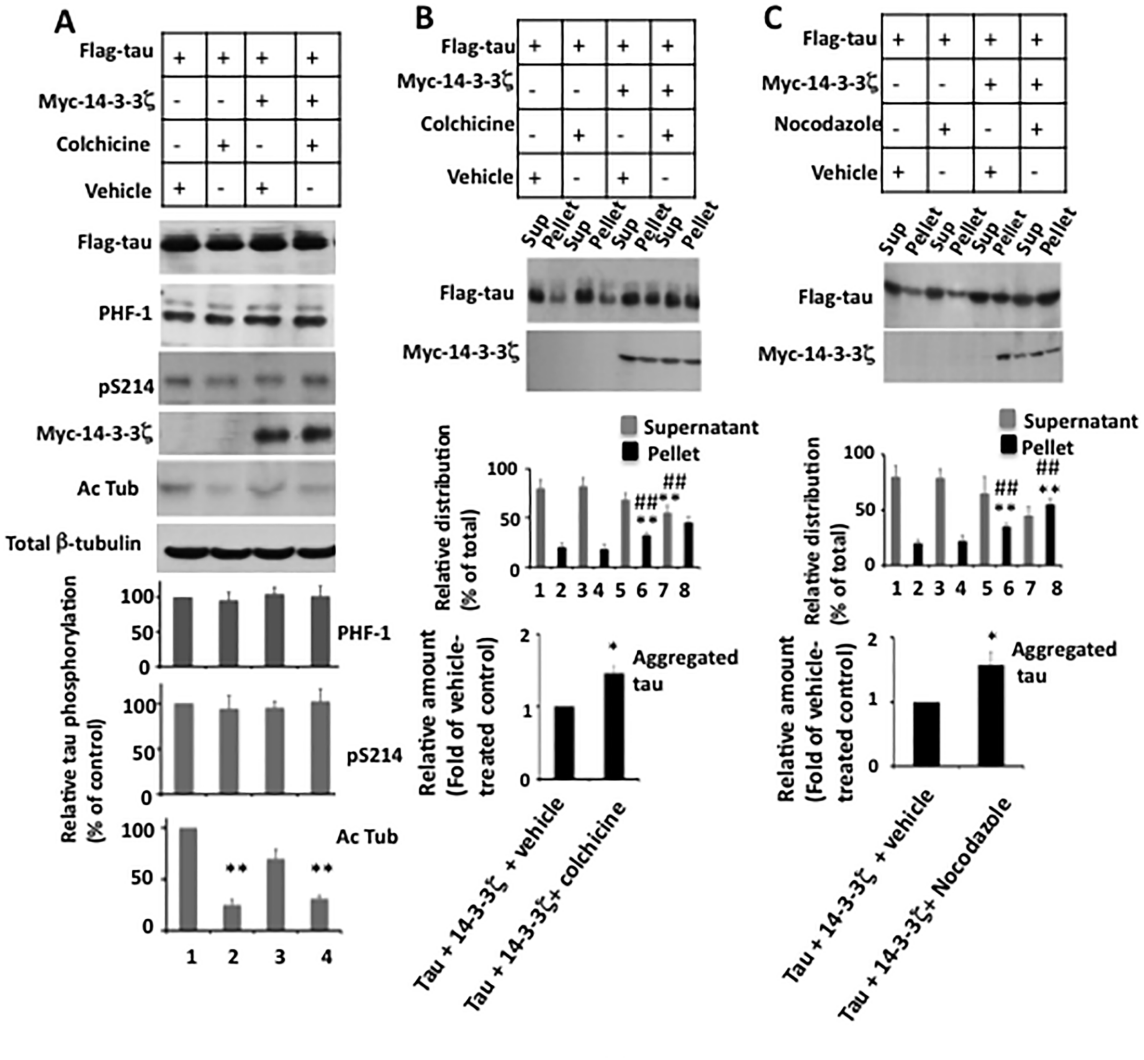
Microtubule disruption promotes 14-3-3ζ-induced tau aggregation in M17 human neuroblastoma cells–M17 human neuroblastoma cells transfected with Flag-tau with or without Myc-14-3-3ζ were treated with indicated microtubule destabilizing drug and then analyzed for tau phosphorylation, microtubule stability and tau aggregation as in Fig 6. *A*, Western blot analysis for tau phosphorylation and microtubule stability. The relative amounts of phosphorylated tau at each site and Ac-Tub were determined as in Figs 4*A* and 6. #*p*< 0.005 against cells transfected with Flag-tau only. *B*, Tau aggregation. Centrifugation assay for tau aggregation of cells treated with the microtubule destabilizing drug colchicine. *C*, Centrifugation assay of cells treated with the microtubule destabilizing drug nocodazole. Relative distribution of Flag-tau in various fractions was calculated as in Fig 4*B*. Values in the bar graph are the average ± S.E. of three experiments. *p> 0.04 against pellet of cells transfected with Flag-tau and Myc-14-3-3ζ and treated with vehicle; **p< 0.05 against the pellet of cells transfected with Flag-tau and treated with vehicle; ##*p* < 0.01 against the pellet of cells transfected with Flag-tau and treated with colchicine or nocodazole.

If the above hypothesis is true, microtubule destabilization independent of tau phosphorylation, should promote 14-3-3ζ-induced tau aggregation. To test this hypothesis, we co-transfected M17 cells with tau and 14-3-3ζ. Transfected cells were treated with either the microtubule destabilizing drug colchicine or vehicle and analyzed for microtubule stability, tau phosphorylation, and tau aggregation. As expected, colchicine did not affect total amount of tubulin but reduced the level of Ac-Tub (Fig 7*A*, lanes 2 and 4). In addition, tau phosphorylation at both the PHF-1 and pS214 sites were similar in cells treated with either colchicine or vehicle. This data confirmed the efficacy of colchicine in disrupting microtubule stability without affecting tau phosphorylation at Ser^396/404^ and Ser^214^ in these cells. However, in cells co-transfected with Flag-tau and Myc-14-3-3ζ and exposed to colchicine, tau aggregation increased by 1.5-fold compared to cells expressing Flag-tau and Myc-14-3-3ζ and exposed to vehicle (Fig 7*B*, *lower panel*). To substantiate this data, we performed a similar experiment where colchicine was substituted with another microtubule destabilizing drug nocodazole. Similar to the colchicine data, a 1.6-fold increase in aggregated tau was observed in cells that were transfected with Flag-tau and Myc-14-3-3ζ followed by exposure to nocodazole, compared to the corresponding control cells that were exposed to vehicle (Fig 7*C*, *lower panel*). These results demonstrated that microtubule destabilization promotes 14-3-3ζ-induced tau aggregation independent of tau phosphorylation in M17 human neuroblastoma cells.

### Microtubules protect tau from 14-3-3ζ–induced aggregation

In microtubule sedimentation assay, tubulin is incubated at 37°C in the presence of GTP/Mg^2+^/taxol. Tubulin polymerizes, forms microtubules, becomes insoluble and can be recovered by centrifugation [33]. If tau is included in the mixture it binds to microtubules and co-sediments with microtubules during centrifugation. When the microtubule pellet is dispersed in cold microtubule depolymerization buffer, microtubules disassemble and become soluble. Microtubule disassembly causes tau to dissociate from microtubules and become soluble [28, 34]. In the tau aggregation assay, tau is incubated with an agent that causes its aggregation and after incubation, the sample is centrifuged. Aggregated tau settles in the pellet and soluble tau remains in the supernatant. The pellet is suspended in the buffer and then centrifuged, and the insoluble tau that remains in the pellet is regarded as the aggregated form [15, 32]. Because of the similarity between the two assays, we slightly modified the microtubule sedimentation assay to evaluate tau aggregation in the presence of microtubules. We incubated tau with 14-3-3ζ in the presence of microtubules/GTP/Mg^2+^/taxol to induce microtubule polymerization. Incubated samples were centrifuged and the pellets were suspended in cold microtubule depolymerization buffer, incubated and centrifuged. If tau became soluble along with the microtubules, it was regarded as the soluble form. If tau did not dissolve, remained in the pellet and was found to display tau positive ultrastructure under EM, it was designated as the aggregated form. In this assay, we used bacterially expressed GST as the control, purified using same procedure as 14-3-3ζ.

**Fig 8:**
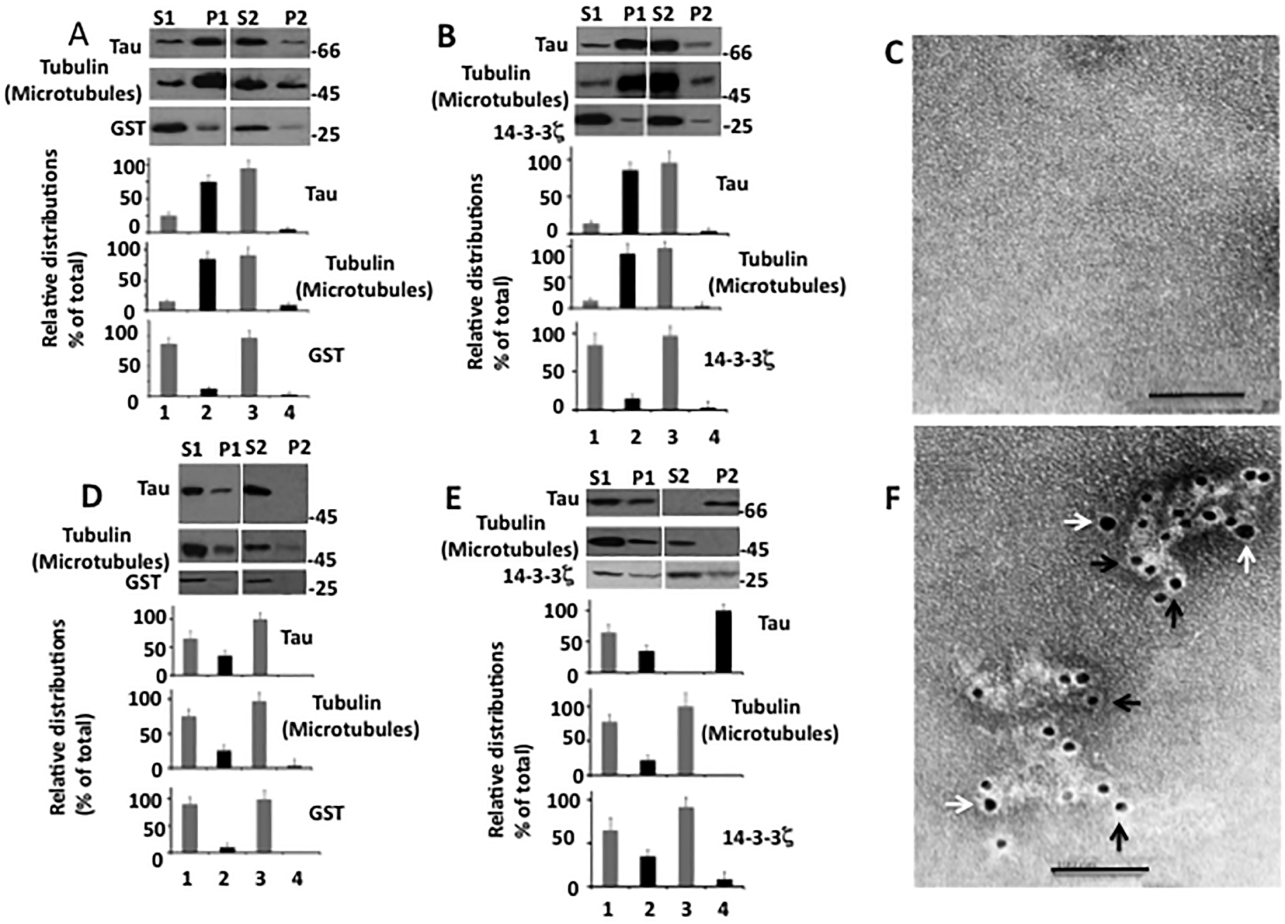
Microtubules protect tau from 14-3-3ζ-induced aggregation *in vitro*–Microtubules sedimentation assay was performed as described in Materials and Methods. *A*, Western blots of microtubule sedimentation assay performed in the presence of GTP/Mg^2+^/taxol followed by the addition of GST. *B*, Western blots of microtubules sedimentation assay in the presence of GTP/ Mg^2+^/taxol followed by the addition of 14-3-3ζ. *C*, Immuno EM of P2 from microtubule sedimentation assay in the presence of GTP/ Mg^2+^/taxol followed by the addition of 14-3-3ζ. *D*, Western blots of microtubule sedimentation assay in the absence of GTP/ Mg^2+^/taxol followed by the addition of GST. *E*, Western blots of microtubule sedimentation assay in the absence of GTP/ Mg^2+^/taxol followed by the addition of 14-3-3ζ. *F*, Immuno EM of P2 from microtubule sedimentation assay in the absence of GTP/ Mg^2+^/taxol followed by the addition of 14-3-3ζ. Scale bars 100 nm.

Microtubules from the sample containing tau, GST, and tubulin were recovered in the pellet P1 after centrifugation (Fig 8*A*, *lane 2*). While tau was present in the pellet P1 (Fig 8*A*, *lane 2*), GST was recovered in the supernatant S1 (Fig 8*A*, *lane 1*). This data showed that microtubule-bound tau settled along with the microtubules, whereas GST did not bind to microtubules and remained in the soluble fraction S1. In the sample containing tau, tubulin, and 14-3-3ζ, microtubules were found in the pellet P1 after centrifugation (Fig 8*B*, *lane 2*) and tau settled along with the microtubules during centrifugation (Fig 8*B*, *lane 2*). On the other hand, 14-3-3ζ did not bind to microtubules and was in the soluble fraction S1 (Fig 8*B*, lane 1).

To determine if tau in the pellet was microtubule-bound or was in an aggregated form, each of the above P1 pellets were resuspended in the microtubule depolymerization buffer, incubated and re-centrifuged. In the control sample containing tau, GST, and tubulin, the microtubules depolymerized and both microtubules and tau were present in the supernatant S2 (Fig 8*A*, *lane 3*). This data determined that tau in the P1 was microtubule-bound and not in an aggregated form. Similarly, in the sample containing tau, tubulin, and 14-3-3ζ, the microtubules also depolymerized and were present in the supernatant S2 (Fig 8*B*, lane 3). Importantly, almost all tau was present in the corresponding supernatant S2 (Fig 8*B*, *lane 3*) and the pellet did not show any tau- or 14-3-3ζ-positive ultrastructure under EM (Fig 8*C*). This data indicated that microtubule-bound tau did not aggregate when incubated with 14-3-3ζ.

In the above experiment, microtubules may have prevented tau from 14-3-3ζ-induced aggregation by acting as a non-specific protein. To rule out this possibility, we performed a similar experiment as described above, but excluded GTP/Mg^2+^/taxol in the microtubule polymerization mixture to prevent microtubule formation. As expected, microtubules were not formed in samples containing tubulin, tau, and GST, as they were present in the supernatant S1 after centrifugation (Fig 8*D*, *lane 1*). Tau from this sample was also present in the supernatant S1 (Fig 8*D*, *lane 1*). Thus, microtubules were not assembled and both microtubules and tau remained soluble in the sample containing tau, GST, and tubulin.

Microtubules were also not formed in the sample containing 14-3-3ζ, tau, and tubulin, as they were found in the corresponding supernatant S1 (Fig 8*E*, *lane 1*). However, ~35% of the total tau was present in the pellet P1 after centrifugation (Fig 8*E*, *lane 2*). Thus, despite the absence of any polymerized microtubules, a significant amount of tau was present in the pellet P1. This data suggested that tau came down into the pellet during centrifugation because it was in its aggregated form. To examine this possibility, the pellet P1 was resuspended in the buffer, incubated and then centrifuged. Most of the tau did not dissolve again and settled in the pellet P2 upon centrifugation (Fig 8*E*, *lane 4*). Under the EM, the P2 displayed large, amorphous ultrastructures that were decorated with both anti-tau (black arrow) and anti-14-3-3ζ (white arrow) gold particles (Fig 8*F*). This data determined that tau in the pellet existed in an aggregated form and that incubation with 14-3-3ζ caused tau aggregation in the presence of unpolymerized microtubules. Since tau binds to only polymerized microtubules [33], this data in turn suggested that microtubule-bound tau is resistant to 14-3-3ζ-induced aggregation.

### Phosphorylation promotes tau aggregation by destabilizing microtubules in vitro

**Fig 9:**
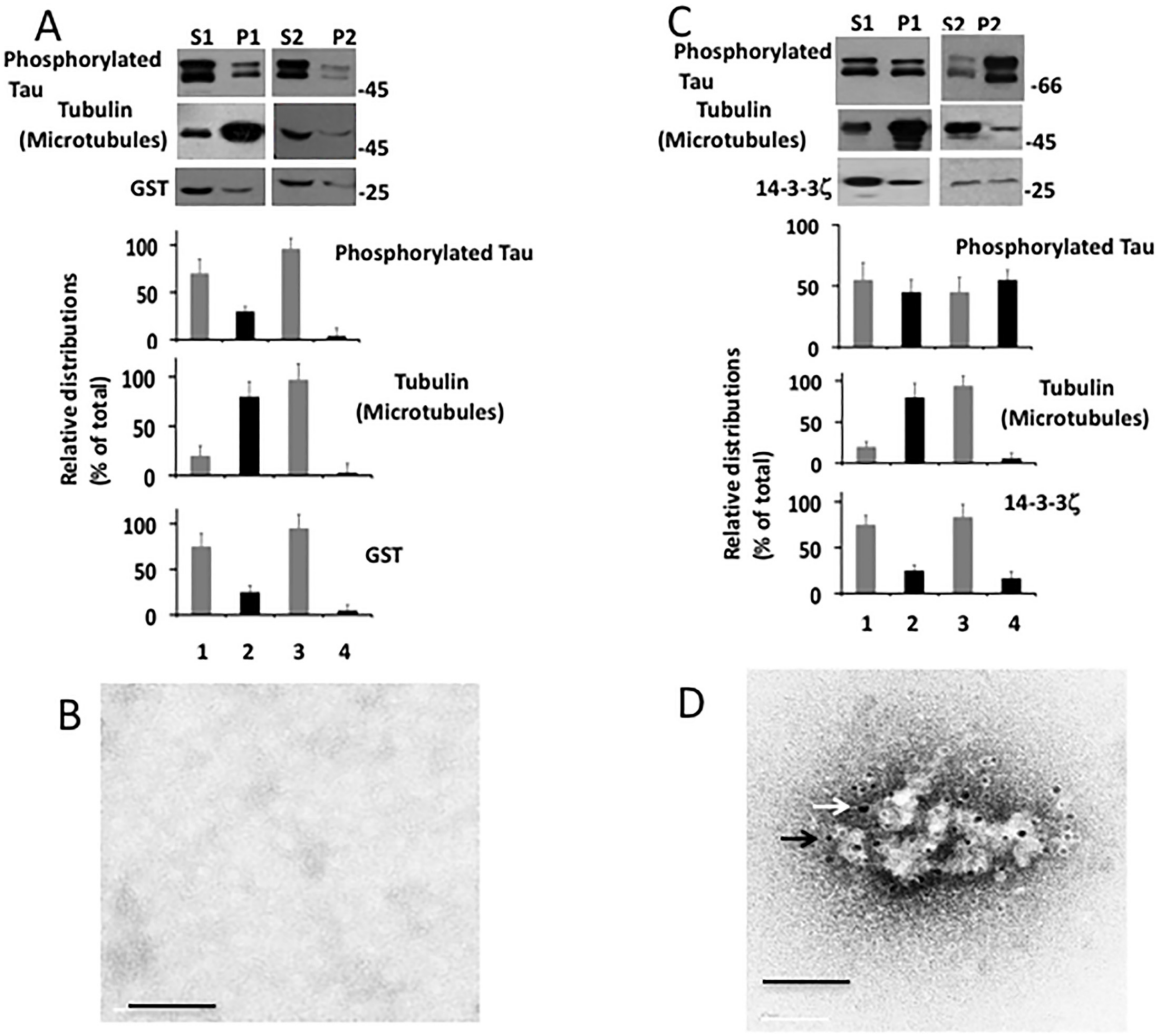
Tau phosphorylation mitigates protective effect of microtubules against 14-3-3ζ-induced tau aggregation. Microtubule sedimentation assay was performed using phosphorylated tau in the presence of GTP/ Mg^2+^/taxol followed by the addition of 14-3-3ζ or GST control as in Fig 8. *A*, Western blot of the samples representing microtubule sedimentation assay performed using GST control. *B*, Immuno EM of P2 from panel *A*. *C*, Western blot of microtubule sedimentation assay performed using 14-3-3ζ. *D*, immuno EM of P2 from panel *C*. Scale bars 200 nm.

If microtubules protect tau from 14-3-3ζ only when tau is microtubule-bound, phosphorylated tau that has significantly reduced affinity for microtubules will be expected to be more susceptible to aggregation even in the presence of polymerized microtubules. To test this possibility, we performed a microtubule sedimentation assay as in Fig 8*A* by using phosphorylated tau. In the sample containing tubulin, GST, and phosphorylated tau, microtubules were formed and were present in the pellet P1 after centrifugation (Fig 9*A*, *lane 2*). GST remained in the supernatant S1 (Fig 9*A*, *lane 1*). Likewise, most of phosphorylated tau was also found in the supernatant S1 (Fig 9*A*, *lane 1*). Thus, phosphorylated tau did not bind to microtubules and remained in the supernatant. In the sample containing phosphorylated tau, tubulin, and 14-3-3ζ, a significant amount of phosphorylated tau was present in the pellet P1 along with microtubules after centrifugation (Fig 9*B*, *lane 2*). To determine if phosphorylated tau in the pellet was in an aggregated or soluble form, pellet P1 was dispersed in the cold microtubule depolymerization buffer, incubated at 4°C and centrifuged. The microtubules depolymerized, became soluble, and were present in the supernatant S2 (Fig 9*B*, *lane 3*). However, most of phosphorylated tau did not dissolve and remained in the pellet P2 (Fig 9*B*, *lane 4*). Under EM, P2 showed amorphous aggregates that were co-labeled with PHF-1 (black arrow), and anti-14-3-3ζ (white arrow) gold particles. This data indicated that phosphorylated tau did not bind to microtubules and aggregated in the presence of 14-3-3ζ. This, in turn, indicated that microtubules do not protect phosphorylated tau from 14-3-3ζ-induced aggregation.

## Discussion

Tau is a soluble protein with very little secondary structure in solution [31], but when incubated with any of the acidic polyanions such as heparin, glutamate, RNA, DNA or fatty acids, it changes conformation and aggregates [6–9]. These polyanions bind to the microtubule-binding repeats of tau that are rich in basic amino acids [8, 11]. NMR studies have shown that polyanions and microtubules share a high degree of binding similarity for tau and that they compete for tau binding. It was suggested that stable microtubules may prevent PHF formation by blocking the polyanion from binding to tau [8].

Like many polyanions that cause tau aggregation, 14-3-3ζ is an acidic molecule and binds to the microtubule-binding region of tau [19, 44]. The presence of 14-3-3ζ in the NFTs of AD brain was reported by several studies [15–17]. It was also noted that 14-3-3ζ was significantly upregulated in AD brain and present in the area of high NFT densities [16]. A number of studies have shown that 14-3-3ζ binds to tau and promotes tau phosphorylation and tau aggregation *in vitro* [18–21, 45–48]. Recently, we demonstrated that 14-3-3ζ is bound to tau in PHFs of AD brain [15]. We also showed that when incubated with 14-3-3ζ *in vitro*, tau forms amorphous aggregates, single-stranded straight filaments, double-stranded ribbon-like filaments, and PHF-like filaments in an incubation-time dependent manner. These filaments display striking resemblance with the corresponding filaments of AD brain [15]. Taken together, these studies suggest that 14-3-3ζ plays a role in the tau fibrillization in AD. Interestingly, phosphorylated and nonphosphorylated tau aggregated in the presence of 14-3-3ζ in a similar manner [15]. This data indicated that tau phosphorylation does not directly influence 14-3-3ζ-induced tau aggregation *in vitro*.

We found that when co-overexpressed with 14-3-3ζ in human M17 neuroblastoma cells, tau forms amorphous aggregates similar to that observed *in vitro* [15]. In contrast to the *in vitro* data however, phosphorylation by PKA or GSK3β promoted 14-3-3ζ-induced tau aggregation (Figs 4 and 5). Moreover, microtubule-disrupting drugs also promoted 14-3-3ζ-induced tau aggregation without promoting tau phosphorylation (Fig 7). *In vitro*, tau aggregated in the presence of 14-3-3ζ when unbound to microtubules (in the presence of unpolymerized tubulin) but became resistant to 14-3-3ζ-induced aggregation when bound to microtubules (Fig 8). Phosphorylated tau on the other hand, did not bind to microtubules and aggregated in the presence of microtubules (Fig 9). A previous study has shown that 14-3-3ζ and microtubules bind tau in a mutually exclusive manner [19]. These results together indicate that microtubules, by binding to tau, mask 14-3-3ζ binding site and thus protect tau from 14-3-3ζ-induced aggregation. Phosphorylation of tau makes it more accessible to 14-3-3ζ by interfering in the binding between tau and microtubules.

PHFs isolated from NFTs display characteristic double stranded fibrillar morphology [49, 50]. In neuroblastoma M17 cells on the other hand, tau forms amorphous aggregate when coexpressed with 14-3-3ζ (Fig 3). Amorphous tau positive aggregates are the first ultrastructures formed in pretangle neurons of AD brain. These amorphous aggregates become fibrillar and form PHFs with the progression of the disease [51]. PHFs contain a number of biological molecules other than tau and 14-3-3ζ [2]. Molecules other than tau and 14-3-3ζ that are expressed in pretangle neurons may be required to drive conversion of amorphous tau aggregates to PHFs.

It is plausible that in the normal brain where most of the tau is not phosphorylated and microtubule-bound, the physiological concentration of 14-3-3ζ is not high enough to cause tau fibrillization. In AD brain, however, as 14-3-3ζ and unbound hyperphosphorylated tau accumulates [16], 14-3-3ζ binds to and causes fibrillization of the accumulated hyperphosphorylated tau. It should be noted that microtubules are less stable in cells overexpressing tau and 14-3-3ζ than in those expressing tau alone (compare lane 2 with lane 1 in Fig 6). The relative amount of aggregated tau also is higher in cells expressing tau and 14-3-3ζ compared to those expressing tau only (Fig 6). Previous studies have shown that 14-3-3ζ binds to tau and promotes tau phosphorylation and its expression is upregulated in AD brain [15, 16, 18, 19, 45, 47, 48]. Increased expression of 14-3-3ζ alone in AD brain therefore may promote tau phosphorylation, destabilization of microtubules and subsequently cause tau aggregation. Our study suggests a novel insight as to how hyperphosphorylated tau forms PHFs in AD brain.

A comprehensive study of human brain detergent insoluble proteome in AD identified ribonucleoprotein U1-70K and other core U1 small nuclear ribonucleoproteins (snRNPs) in NFTs [52]. This study also showed a significant defect in RNA maturation caused by aggregation of ribonucleoproteins in AD brain. Interestingly, *in vitro* U1-70K aggregation was induced by aggregated protein (s) of AD brain and this aggregation did not correlate with level of tau. This data suggested that protein (s) other than tau causes U1-70K aggregation [53]. 14-3-3ζ is a dimeric scaffolding protein and can bind and bring two different proteins together [47]. 14-3-3ζ binds to tau [19] and a number of nuclear proteins [44, 54] and is a component of NFTs [15, 17]. It will be interesting to examine if 14-3-3ζ simultaneously binds to and brings U1-70K and tau to NFTs in AD brain.

## Supporting Information

S1 Fig Tau and 14-3-3ζ co-localize in human neuroblastoma M17 cells. M17 cells co-transfected with Flag-tau and Myc-14-3-3ζ were fixed and immunofluorescent images were captured. Flag-tau (red), Myc-14-3-3ζ (green) and co-localization (yellow) are shown. Scale bar, 100 μM.
(TIF)

S2 Fig Western blot of M17 neuroblastoma cells transfected with Flag-tau and Myc-14-3-3ζ. Cell lysates were Western blotted against indicated antibodies to monitor levels of tau and 14-3-3ζ. Tau 5 recognizes both endogenous tau and Flag-tau. Likewise, anti-14-3-3ζ antibody is immunoreactive against both endogenous 14-3-3ζ and Myc-14-3-3ζ. These cells express low levels of endogenous tau (lane 2) and 14-3-3ζ(lane 1).
(TIF)
