# Supplementary figures and images for: 14-3-3ζ Mediates Tau Aggregation in Human Neuroblastoma M17 Cells
Source: PLoS One. 2016 Aug 22;11(8):e0160635. doi: 10.1371/journal.pone.0160635 (PMC4993442; doi:10.1371/journal.pone.0160635)

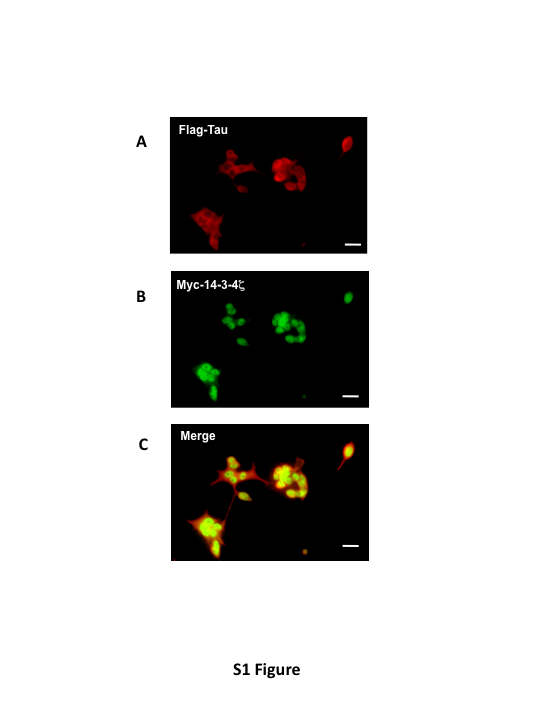

Supplement: S1 Fig — M17 cells co-transfected with Flag-tau and Myc-14-3-3ζ were fixed and immunofluorescent images were captured. Flag-tau (red), Myc-14-3-3ζ (green) and co-localization (yellow) are shown. Scale bar, 100 μM. (TIF) [file pone.0160635.s001.tif]

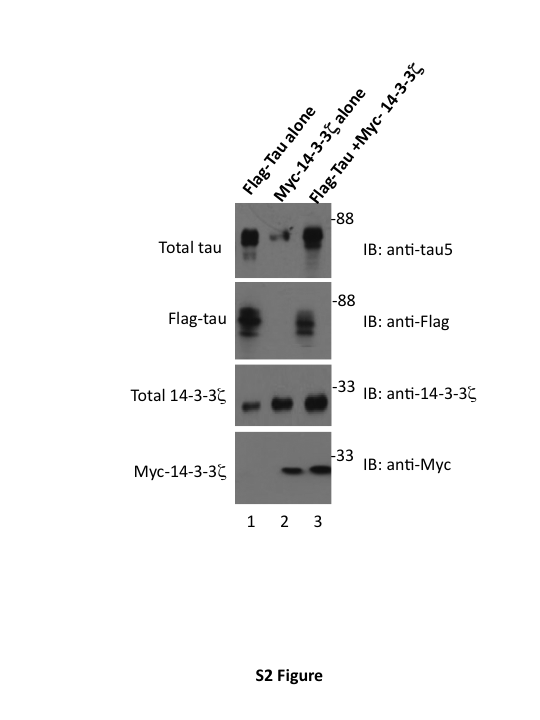

Supplement: S2 Fig — Cell lysates were Western blotted against indicated antibodies to monitor levels of tau and 14-3-3ζ. Tau 5 recognizes both endogenous tau and Flag-tau. Likewise, anti-14-3-3ζ antibody is immunoreactive against both endogenous 14-3-3ζ and Myc-14-3-3ζ. These cells express low levels of endogenous tau (lane 2) and 14-3-3ζ(lane 1). (TIF) [file pone.0160635.s002.tif]
